# Clinical Outcomes of Aspirin and Clopidogrel among Patients with Chronic Obstructive Lung Disease: Insights from a Meta-Analysis

**DOI:** 10.3390/jcm13133715

**Published:** 2024-06-26

**Authors:** Naif M. Alhawiti, Taha T. Ismaeil, Sherouk Fouda, Badi A. Alotaibi, Ashraf El-Metwally, Tlili Barhoumi, Tareq F. Alotaibi

**Affiliations:** 1 Department of Clinical Laboratory Sciences, College of Applied Medical Sciences, King Saud bin Abdulaziz University for Health Sciences, Riyadh 11481, Saudi Arabia; 2 King Abdullah International Medical Research Center, Riyadh 21423, Saudi Arabia; ismaeilt@ksau-hs.edu.sa (T.T.I.); barhoumitl@ngha.med.sa (T.B.); alotaibita@ksau-hs.edu.sa (T.F.A.); 3 Department of Respiratory Therapy, College of Applied Medical Sciences, King Saud bin Abdulaziz University for Health Sciences, Riyadh 11481, Saudi Arabia; 4 Department of Respiratory Care, King Abdulaziz Medical City, Riyadh 21423, Saudi Arabia; 5 College of Biomedical Sciences, RMIT University, Melbourne, VIC 3000, Australia; sheroukf@gmail.com; 6 College of Public Health and Health Informatics, King Saud bin Abdulaziz University for Health Sciences, Riyadh 11481, Saudi Arabia; ashraf.elmetwally@gmail.com; 7 Medical Research Core Facility and Platforms, King Saud bin Abdulaziz University for Health Sciences, Riyadh 11481, Saudi Arabia

**Keywords:** aspirin, clopidogrel, clinical outcomes, COPD, meta-analysis

## Abstract

(1) Background: Aspirin and clopidogrel have been found helpful in improving clinical outcomes among patients with chronic obstructive lung disease (COPD). However, the evidence on the efficacy of aspirin and/or clopidogrel on clinical outcomes has not been synthesized and summarized in the prior reviews. Hence, we undertook a meta-analysis of the research studies examining the effect of aspirin and/or clopidogrel on varying clinical outcomes among COPD patients; (2) Methods: Using key search terms, we searched databases, including MEDLINE, CINAHL, Google Scholar, and EMBASE to find observational studies and RCTs. Our search was limited to research written in English. We used a random effect model to calculate the 95% confidence intervals and pooled hazard ratio; (3) Results: We included 12 eligible research studies (33,8008 patients) in the current meta-analysis. Among COPD patients, the hazard of all-cause mortality among users of aspirin or clopidogrel was 17% lower (HR: 0.83; 95% CIs (0.70, 0.97; I^2^ = 73%, X2: 33.34) compared to non-users of anticoagulants (aspirin or clopidogrel). The hazard of dyspnea among users of aspirin or clopidogrel was 3% lower (HR: 0.97; 95% CIs (0.27, 3.49; I^2^ = 93%, X2: 42.15) compared to non-users of anticoagulants (aspirin or clopidogrel). There was no statistically significant effect of aspirin on other clinical outcomes such as myocardial infarction (HR: 2.04; 95% CIs (0.02, 257.33) and major bleeding (HR: 1.93; 95% CIs (0.07, 1002.33). The funnel plot and Egger’s regression test did not show any evidence of publication bias; (4) Conclusions: Overall, we found a positive and beneficial effect of aspirin and/or clopidogrel in reducing all-cause mortality among COPD patients. However, there is uncertainty of evidence for other clinical outcomes such as exacerbation of dyspnea, myocardial infarction, and major bleeding. A limited number of studies examining other clinical outcomes warrant conducting more robust epidemiological studies to assess the efficacy and safety of aspirin and clopidogrel on other clinical outcomes among COPD patients.

## 1. Introduction

Chronic obstructive lung disease (COPD) is a type of progressive lung pathology that is characterized by compromised lung function and chronic bronchitis [1]. COPD is one of the major causes of disability and is ranked as the third leading cause of mortality worldwide [1,2]. Moreover, COPD is a widely prevalent global health issue owing to its high prevalence (approximately 10%), increasing incidence and burden, and associated social and economic costs [3]. More than three-quarters of the worldwide COPD cases are in low–middle-income countries, and health systems worldwide are putting population-wide efforts to bring reforms in resource-poor settings to address the burden of COPD [4]. Given the importance of COPD, the Global Initiative for Chronic Obstructive Lung Disease (GOLD) has organized World COPD Day to raise public awareness and improve COPD care across the globe [3]. The pharmacologic treatment of COPD mainly consists of varying combinations of inhalers aimed at reducing bronchiole inflammation and improving bronchodilation [5]. Nevertheless, COPD manifests in a variety of ways, including but not limited to systematic inflammation, which is perhaps not adequately addressed by present regimes. For example, corticosteroid inhalers have not been successful in decreasing the inflammatory biomarkers [6]. Similarly, another line of therapy for COPD includes chronic macrolide antibiotics, which reduce inflammation and possess immunomodulatory characteristics along with antibacterial properties [7]. While this regime has also been found to be helpful in reducing acute exacerbations among patients with severe COPD, the use of chronic macrolide antibiotics is limited due to the risk of antibiotic resistance and substantial hearing loss among users [8,9]. Finally, another commonly used therapy for COPD is phosphodiesterase-4 inhibitor roflumilast, which decreases the rate of exacerbation but only among patients with chronic bronchitis and severe COPD [10]. However, due to its gastrointestinal side effects, the use of this therapy is also limited [11]. Statins, which are believed to possess anti-inflammatory features and decrease C-reactive protein, are found to be ineffective in decreasing the rate of exacerbation among patients with moderate to severe COPD [12].

Given the above-mentioned limitations of the existing pharmacological therapy for COPD, clinicians and researchers have started using anticoagulant therapy such as aspirin and clopidogrel. Aspirin inhibits the activity of platelets by inhibiting the conversion of arachidonic acid, and aspirin has both direct and indirect anti-inflammatory properties [13]. These properties have been found helpful to improve clinical outcomes such as reducing the rate of exacerbation of dyspnea, length of stay, and overall mortality among COPD patients [14,15]. The odds of mechanical ventilation among COPD patients are also reduced with the use of aspirin and clopidogrel [16]. However, the evidence on the efficacy of aspirin and/or clopidogrel on clinical outcomes has not been synthesized and summarized in the prior reviews. To address this gap in knowledge, we undertook a meta-analysis of the research studies, examining the effect of aspirin and/or clopidogrel on varying clinical outcomes among COPD patients. The rationale for conducting the current meta-analysis was to provide useful insights into the utility of aspirin or clopidogrel in reducing soft outcomes, such as dyspnea and major bleeding, as well as hard outcomes, including all-cause mortality among patients diagnosed with COPD. We hypothesized that, given the previously established biological anti-inflammatory properties of aspirin and clopidogrel, these two medications substantially reduced the hazard of adverse outcomes, including all-cause mortality, dyspnea, myocardial infarction, and length of stay. The findings of this meta-analysis may help researchers, clinicians, and policy-makers to develop guidelines for the use of aspirin and clopidogrel among COPD patients.

## 2. Materials and Methods

### 2.1. Selection of Studies and Data Sources

Articles were searched using databases such as PubMed, EMBASE, and Google Scholar. We used key search terms such as “aspirin”, “clopidogrel”, “anticoagulant”, “aspirin or clopidogrel”, “COPD”, “Chronic obstructive pulmonary disease”, “clinical outcomes”, “length of hospital stay”, “mortality”, “dyspnea”, “exacerbation of COPD symptoms”, “major bleeding”, “stroke”, and “myocardial infarction”. We used Boolean operators such as “OR” and “And” to combine the above-mentioned search terms.

### 2.2. Eligibility Criteria

All research studies that examined clinical outcomes such as cardiovascular events, length of hospital stay, major bleeding and/or stroke, dyspnea or its exacerbation, and all-cause mortality were included in this review. To include a wide range of articles, we did not apply restrictions to the type of outcomes; rather, we aimed to include all types of outcomes studied by authors of the primary study. Given the scarcity of the literature on this topic, we also did not apply restrictions on study design and included all types of quantitative studies, such as randomized controlled trials, cohort studies, case-control studies, and cross-sectional studies. However, we did not include qualitative studies, case reports, commentaries, the grey literature, reviews, and letters to the editors. We included research papers in the meta-analysis to see if they had reported the sample size or number of clinical events of a particular outcome in the intervention/exposure and control groups. Studies with insufficient information to compute the pooled summary measures and their respective confidence intervals were excluded. Due to the paucity of the literature on the given topic, we did not apply limits to time and year of publication; however, we limited our search to only articles published in the English language. We also gathered data from the bibliographies of full-text eligible papers. The review comprised the relevant papers from the list of references.

### 2.3. Data Extraction 

The reviewers extracted the data using a pre-defined data extraction tool. The year, the nation, the sample size, the number of clinical events in the intervention and control arms, the type of outcomes measured, the study design, the study name, and the author were among the data the authors retrieved. First, abstracts were analyzed, and then, full-text publications were examined to determine whether or not they could be included in the meta-analysis. After obtaining data on the aforementioned parameters, we created a database using the Excel sheet. Along with the sample size of each trial, we also gathered data according to the types of outcomes and recorded the number of clinical events in the exposure and control arms according to the types of outcomes.

### 2.4. Statistical Methods

We used information on clinical events and the total number of research participants from both groups for each outcome. The inverse of variance method was used to assign weights to each study based on the sample size, which was used to compute the variance of each study. Based on the inverse of variance, weight was assigned to each study. Because we anticipated heterogeneity among research, we used a random effect model that contained data from both inside and across studies, and we did not assume the common true study measure across all studies. We used I^2^ statistics and reported a chi-square test with the degree of freedom and the *p*-value of the heterogeneity statistics to evaluate heterogeneity. I^2^ statistics of 0–50% were considered indicative of low heterogeneity, 50–75% indicated moderate heterogeneity, and I^2^ statistics greater than 75% indicated substantial heterogeneity across the studies. The funnel plot was created to show the overall risk of different outcomes for both the exposed and unexposed groups, as well as the pooled hazard ratio with 95% confidence intervals. The funnel-plot-based method is a type of visual examination of a funnel plot that presents effect sizes plotted against respective standard errors. In the presence of publication bias, the funnel plot will be skewed. We confirmed the publication bias by employing Egger’s regression asymmetry test, a quantitative and more objective test, to assess publication bias to supplement funnel plot visual asymmetry. Egger’s test regresses the standardized study effect sizes on their precision, and the regression intercept is expected to be zero in the absence of publication bias. Using Egger’s regression asymmetry test and a funnel plot to assess plot symmetry, we investigated publication bias. R 4.2.2 software was used for analysis [17].

### 2.5. Tools to Assess Quality of Studies

Because the current meta-analysis included papers with a variety of study designs, relying on a single tool or method to assess study quality is inappropriate. As a result, we used two separate tools, including the Cochrane risk-of-bias tool for RCTs (RoB 2.0) and the Newcastle–Ottawa Scales for observational studies.

## 3. Results

### 3.1. Search Strategy

**Figure 1:**
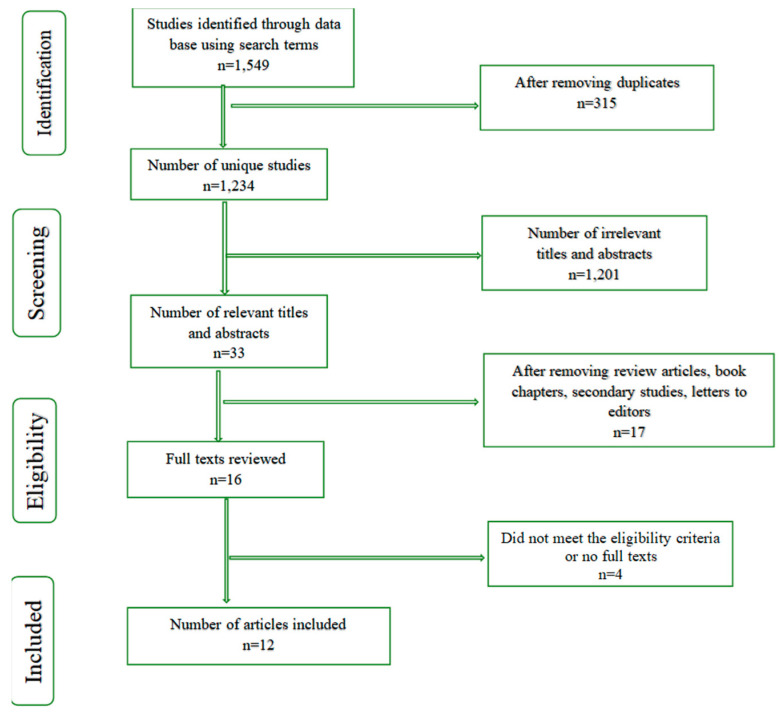
Flow chart summarizing the identification and selection of studies included in the quantitative synthesis for meta-analysis.

Initially, screening started with reviewing titles and abstracts. Then, the reviewers read and evaluated the full-text articles for eligibility. First, 1549 research articles were identified by searching various electronic databases. We removed 315 duplicate records. As a result, 1234 unique studies were identified. Further, we excluded 1201 articles due to irrelevant titles and abstracts. Hence, we found 33 eligible studies after screening titles and abstracts. Because of the reasons illustrated in Figure 1, we removed an additional 17 studies. Thus, 16 full-text articles were read and reviewed. Later, two additional records were removed because they did not meet the eligibility criteria for the meta-analysis. Hence, 12 studies were included in the meta-analysis, as shown in the PRISMA flow diagram (Figure 1).

### 3.2. Features of the Identified Research Studies

Table 1 shows the features of the observational studies and randomized controlled trials (RCTs) identified for the meta-analysis (*n* = 12 research studies; 338,008 patients). It appears that all studies were very recent and were published in the past one and half decades between January 2007 and January 2023. Out of 12 citations, two were RCT, and 10 were observational studies, including both retrospective and prospective cohort studies. One study was multi-country; one study each was undertaken in Norway, Scotland, the United Kingdom (UK), Italy, and Spain. Two studies each were published in China, the United States, and Sweden. The sample size of eligible studies was between 46 and 206,686 patients, as shown in Table 1. The main intervention was aspirin in the majority of the research studies; however, one study offered clopidogrel, and one study assessed the effect of both aspirin and clopidogrel. The duration of the follow-up of all studies varied in length between one month and more than 4 years. Overall, the authors of the identified studies aimed to assess the clinical outcomes of aspirin and/or clopidogrel among COPD patients. However, upon data extraction, the most commonly studied outcome was all-cause mortality (*n* = 10), followed by exacerbation of dyspnea (*n* = 4), major bleeding (*n* = 2), and myocardial infarction (*n* = 2) (Table 1).

### 3.3. Main Findings of Each Study

Soyseth et al., 2007, undertook a hospital-based study in Norway on 854 patients with COPD who were either prescribed aspirin or not prescribed aspirin [18]. The authors studied all-cause mortality as an outcome of interest and found that the hazard ratio of all-cause mortality among users of aspirin was 0.57 (95% CI: 0.58–0.98) after adjusting for age, sex, smoking, pulmonary function, and other co-morbidities [18]. After three years, Short et al., 2011, published a hospital-based study from Scotland on 5977 COPD patients with an intervention duration of 4.35 years [19]. The authors found a 22% reduction in all-cause mortality following the use of beta blockers independently of using other drugs or cardiovascular diseases [19]. Ekstrom et al., 2013, reported data from 2249 patients in Sweden following time-dependent analysis [14]. The authors found that after using aspirin for 1.1 years, there was a 14% reduction in all-cause mortality; however, the results were not statistically significant [14]. In 2014, Harrison published data from an observational cohort study on 1342 COPD patients [15]. Patients took aspirin for about 1.1 years, and authors found about a 40% reduction in all-cause mortality with statistically significant results (HR: 0.63; 95% CI: 0.51, 0.79) [15]. Contrary to these findings, Andell et al., 2015, conducted a multi-country double-blinded controlled trial on 1085 patients diagnosed with COPD and acute coronary syndrome [20]. The authors found that compared to Ticagrelor, there was a higher hazard for all-cause mortality by about 51% (HR: 1.51; 95% CI: 1.10, 2.07) [20]. However, there was a reduction in dyspnea by about 45% (HR: 0.55; 95% CI: 0.41, 0.74) in the same study but a higher hazard of major bleeding (HR: 1.17; 95% CI: 0.84, 1.62) by 17% and of myocardial infarction (HR: 1.37; 95% CI: 0.92, 2.03) by 37% [20]. In 2017, there was an RCT conducted by Campo et al. in Italy on 46 patients with an acute exacerbation of COPD. The authors found a significant reduction in the hazard of all-cause mortality by about 30% (HR: 0.71; 95% CI: 0.60, 0.84) and about 80% reduction in dyspnea (HR: 0.18; 95% CI: 0.01, 4.03), albeit with statistically non-significant results [21]. Gote et al. reported a retrospective cohort study from the United States of America on a relatively large sample size of 206,686 patients with an acute exacerbation of COPD. The authors found a significant reduction in the hazard of all-cause mortality by about 30% (HR: 0.71; 95% CI: 0.60, 0.84) [22]. Fawzy et al., 2018, published study findings from 1698 COPD patients who used aspirin for about one month [23]. The authors found that there was a non-significant reduction in the hazard of dyspnea by about 12% (HR: 0.98; 95% CI: 0.79, 1.21) [23]. Rodríguez-Mañero et al., 2019 undertook a prospective analysis in Spain on 937 patients with COPD [24]. The authors documented a statistically significant reduction in all-cause mortality by about 35% (HR: 0.64; 95% CI: 0.49, 0.83) [24]. In the following year, Ellingsen et al., 2020, published results from an observational retrospective cohort study on 17,745 COPD patients who consumed aspirin for about 3.62 years [25]. The authors noticed a reduction in the hazard of all-cause mortality by about 12% (HR: 0.64; 95% CI: 0.49, 0.83) [25]. Yu et al., 2022, studied 42,728 COPD patients from China who used aspirin for 2.6 years. The authors found a reduction in all-cause mortality by about a quarter (HR: 0.76; 95% CI: 0.66, 0.87) [26]. However, authors reported that a hazard of major bleeding was 3.12 times more likely among aspirin users than non-users (HR: 3.12; 95% CI: 2.96, 3.28) [26]. Lastly, very recently, in 2023, Li et al., 2023, undertook a retrospective cohort study on 56,660 COPD patients in China [27]. The authors found a significant reduction in mortality by about 11% (HR: 0.89; 95% CI: 0.65, 1.22) but with a high hazard of dyspnea and myocardial infarction by a factor of 2.30 (HR: 2.30; 95% CI: 1.67, 3.17) and 2.93, respectively (HR: 2.93; 95% CI: 2.28, 3.77) [27]. 

### 3.4. Forest Plots: Clinical Outcomes

**Figure 2:**
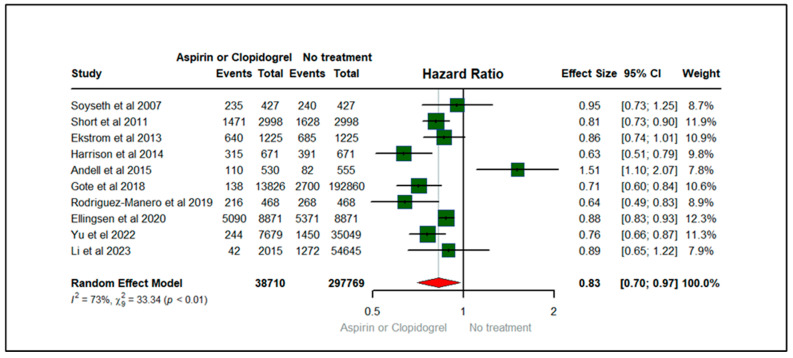
Forest plot illustrating the effect of aspirin or clopidogrel (anticoagulants) on all-cause mortality among patients with COPD [14,15,18,19,20,22,24,25,26,27].

Figure 2 demonstrates the forest plot depicting the effect of aspirin or clopidogrel (anticoagulants) on all-cause mortality among patients with COPD. There were 10 studies that contributed to all-cause mortality, published between 2007 and 2023. The total sample size of 10 studies was 336,479. Among COPD patients, the hazard of all-cause mortality among users of aspirin or clopidogrel was 17% lower (HR: 0.83; 95% CIs (0.70, 0.97) compared to non-users of anticoagulants (aspirin or clopidogrel). The heterogeneity statistics (I^2^ = 73%, X2: 33.34) suggest a reasonable heterogeneity across the studies with statistically significant *p*-value (*p* < 0.01), implying that variability between studies was high for the studies evaluating all-cause mortality, as shown in Figure 2.

**Figure 3:**
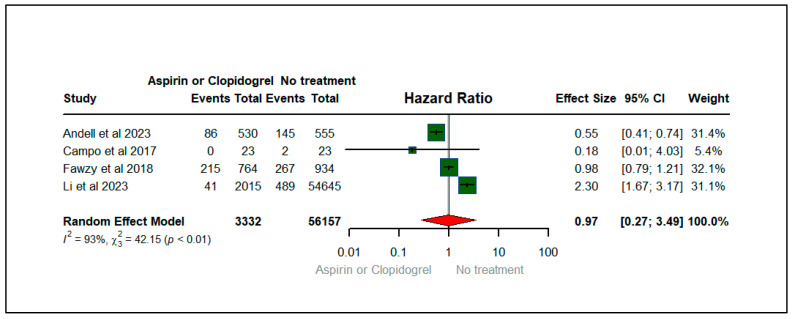
Forest plot illustrating the effect of aspirin or clopidogrel (anticoagulants) on exacerbation of dyspnea among patients with COPD [20,21,23,27].

Figure 3 shows the forest plot depicting the effect of aspirin or clopidogrel (anticoagulants) on the exacerbation of dyspnea among patients with COPD. There were four studies that contributed to the exacerbation of dyspnea, published between 2017 and 2023. The total sample size of the four studies was 59,489. Among COPD patients, we found that the hazard of dyspnea among users of aspirin or clopidogrel was 3% lower (HR: 0.97; 95% CIs: 0.27, 3.49) compared to non-users of anticoagulants (aspirin or clopidogrel). However, the results were not statistically significant. The heterogeneity statistics (I^2^ = 93%, X2: 42.15) suggest a reasonable heterogeneity across the studies with a statistically significant *p*-value (*p* < 0.01), implying that variability between studies was high for the studies evaluating dyspnea, as illustrated in Figure 3.

**Figure 4:**
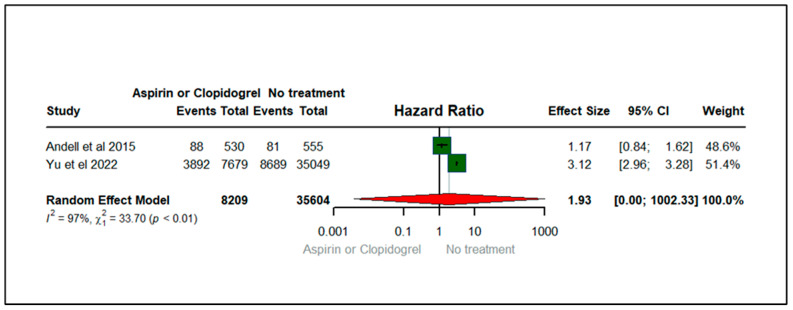
Forest plot illustrating the effect of aspirin or clopidogrel (anticoagulants) on major bleeding among patients with COPD [20,26].

Figure 4 shows the forest plot illustrating the effect of aspirin or clopidogrel (anticoagulants) on major bleeding among patients with COPD. There were two studies that contributed to the exacerbation of dyspnea, published between 2015 and 2022. The total sample size of the two studies was 43,813. Among COPD patients, we found that the hazard of major bleeding among users of aspirin or clopidogrel was 93% higher (HR: 1.93; 95% CIs: 0.07, 1002.33) compared to non-users of anticoagulants (aspirin or clopidogrel). Nevertheless, the results were not statistically significant. The heterogeneity statistics (I^2^ = 97%, X2: 33.7) suggest a reasonable heterogeneity across the studies with a statistically significant *p*-value (*p* < 0.01), implying that variability between studies was high for the studies evaluating major bleeding, as illustrated in Figure 4.

**Figure 5:**
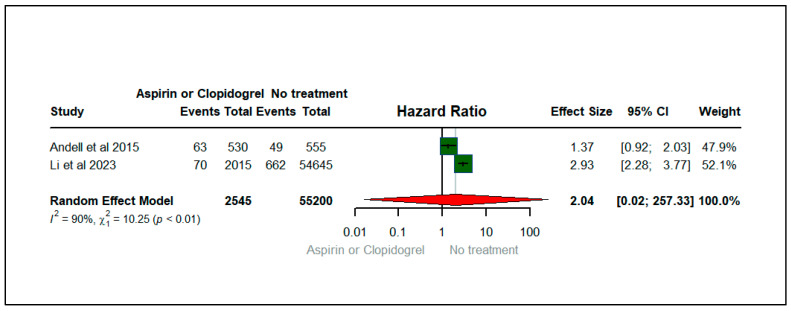
Forest plot illustrating the effect of aspirin or clopidogrel (anticoagulants) on myocardial infarction among patients with COPD [20,27].

Figure 5 shows the forest plot illustrating the effect of aspirin or clopidogrel (anticoagulants) on myocardial infarction among patients with COPD. There were two studies that contributed to myocardial infarction, published between 2015 and 2023. The total sample size of the two studies was 57,745. Among COPD patients, we found that the hazard of myocardial infarction was two times higher among (HR: 2.04; 95% CIs (0.02, 257.33)) aspirin or clopidogrel users than non-users of anticoagulants (aspirin or clopidogrel). Nevertheless, the results were not statistically significant. The heterogeneity statistics (I^2^ = 90%, X2: 10.25) suggest a reasonable heterogeneity across the studies with a statistically significant *p*-value (*p* < 0.01), implying that variability between studies was high for the studies evaluating myocardial infarction, as illustrated in Figure 5.

### 3.5. Results of Publication Bias

**Figure 6:**
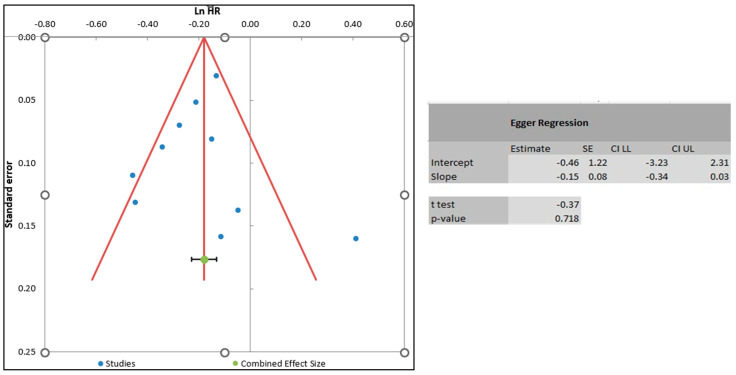
Funnel plot and Egger’s regression test showing evidence of publication bias.

Figure 6 below shows the publication bias of studies examining all-cause mortality. The evidence of publication bias was less likely, as illustrated by the symmetrical shape of the funnel plot in Figure 6. The evidence of a symmetrical funnel plot was endorsed by Egger’s regression test. The findings of Egger’s regression test demonstrated a *p*-value of 0.72, suggesting that the likelihood of publication bias was less likely. Due to very few studies on other outcomes (*n* < 10), including major bleeding, dyspnea, and myocardial infarction, publication bias was not measured for those outcomes.

## 4. Discussion

This meta-analysis was conducted to assess the efficacy of aspirin and clopidogrel on clinical outcomes among patients with COPD. The findings of the studies revealed that aspirin and clopidogrel appeared to improve the survival of COPD patients by reducing the risk of mortality. While there is evidence that aspirin and clopidogrel may reduce the exacerbation of dyspnea among COPD patients, the findings are not statistically significant. Additionally, there is uncertainty of evidence regarding the role of aspirin and/or clopidogrel in improving other outcomes, such as reducing the risk of myocardial infarction and major bleeding.

Our findings are consistent with prior reviews of five observational studies, where authors found that aspirin reduced the risk of all-cause mortality among COPD patients by about 19% [28]. However, the evidence from the current meta-analysis and prior review on the effect of aspirin or clopidogrel in reducing mortality among COPD patients is mainly driven by data on aspirin. Hence, further studies are required to assess the effectiveness of clopidogrel in improving clinical outcomes among COPD patients. 

Aspirin seems to have both local pulmonary and systematic modes of action that may explain the findings of the current meta-analysis. The potential mechanism by which aspirin improves the survival of COPD patients is by impeding the inflammatory pathways subsequent to the activation of platelets. Among COPD patients, the activation of platelets causes microvascular thrombosis, which contributes to organ ischemia and damage of tissues [29]. Aspirin use plays a role in reducing the inflammation related to platelet activation and preventing microvascular thrombus formation by impeding the expression of adhesion molecules [29]. Similar benefits of aspirin have been found in other systematic inflammatory conditions such as sepsis and acute respiratory syndrome [30]. 

Additionally, the evidence suggests that activated platelets secrete a urinary metabolite of thromboxane A_2_, which is elevated among COPD patients [31]. Since aspirin blocks the activity of platelets, it will reduce the production of the metabolite irreversibly [32]. Moreover, in a few other patients, it has been found that aspirin attenuated the production of C-reactive proteins and inflammatory marker IL-6, which might manifest a systematic inflammatory phenotype of COPD [33]. Aspirin has also been found to reduce the production of cytokines among the population of healthy volunteers [34]. Similar to aspirin, clopidogrel is an adenosine diphosphate P2Y12 receptor antagonist that inhibits platelet aggregation after binding to the P2Y12 receptor [35]. However, clopidogrel is considered an irreversible indirect inhibitor that needs to be transformed into its active form before binding to the P2Y12 receptor to inhibit platelet aggregation [35]. These mechanisms may collectively explain why aspirin may prove beneficial in improving survival among COPD patients. 

### 4.1. Strengths and Limitations

There are several strengths of the current meta-analysis. Because it included both observational studies and RCTs without imposing any restrictions on the year of publication, this meta-analysis was unlike any other of its sort. Rather than offering a narrative, we conducted a quantitative summary of this review to offer valuable insights into the effect of aspirin and/or clopidogrel in improving clinical outcomes among COPD patients. The forest plots’ symmetrical shapes demonstrated that there was no publication bias, indicating that small study effects had no bearing on the results of our investigation. Moreover, this meta-analysis provides evidence that may be useful for policy-makers and clinicians to make informed decisions regarding the role of aspirin in reducing mortality and dyspnea among COPD patients.

However, the current meta-analysis has some limitations. First, the evidence regarding the positive effect of aspirin in reducing mortality among COPD patients is mainly driven by observational studies. Observational studies are challenged by issues of unmeasured confounding and measurement error in exposure. The use of aspirin in observational studies is mainly driven by patient characteristics and risk factors. Moreover, the issue of unmeasured confounding cannot be avoided in observational studies. Hence, the findings of the current meta-analysis need to be interpreted cautiously. However, limited evidence from RCTs suggests that future robust epidemiological studies are required to confirm the benefit of aspirin in improving clinical outcomes. Second, the use of aspirin is mainly self-reported in observational studies; therefore, the likelihood of measurement error in exposure remains. Furthermore, due to the unavailability of data on the dosage of aspirin, it is difficult to conclude any dose-response relationship between aspirin and clinical outcomes among COPD patients. Also, the eligible studies mainly considered aspirin, not clopidogrel. Therefore, the evidence regarding the beneficial effects of clopidogrel on clinical outcomes is not clear. Hence, future studies with large sample sizes are warranted to confirm the role of aspirin and clopidogrel in improving clinical outcomes among patients with COPD.

### 4.2. Clinical Implications and Future Directions

The findings of the current meta-analysis provide useful insights into the utility of anticoagulants such as aspirin or clopidogrel. Overall, favorable positive effects of aspirin or clopidogrel in reducing all-cause mortality suggest the use of these medications in the future among COPD patients. Although we found a beneficial effect of aspirin or clopidogrel in reducing all-cause mortality with consistent findings from a majority of the observational studies, there was one RCT with the opposite findings. While RCTs are considered gold standards with respect to study designs, inverse findings from one small RCT could be due to chance alone; therefore, they need to be interpreted cautiously. Also, findings from some studies suggested an inverse effect of aspirin or clopidogrel on major bleeding or myocardial infarction. However, since not all studies reported findings on these intermediate outcomes, it was challenging for us to make a firm conclusion. Hence, more robust epidemiological studies, preferably RCTs, should be designed in the future to confirm the findings of the current meta-analysis. In addition, since there were very few studies that reported the beneficial effects of clopidogrel, there is a need to conduct more studies in the future to assess the effect of clopidogrel on all-cause mortality, dyspnea, length of stay, and major bleeding among COPD patients. 

## 5. Conclusions

Overall, we discovered that using aspirin and/or clopidogrel helped reduce all-cause mortality in COPD patients. Other clinical outcomes, such as worsening dyspnea, myocardial infarction, and severe bleeding, have inconsistent results. More extensive epidemiological studies are required to assess the efficacy and safety of aspirin and clopidogrel on other clinical outcomes among COPD patients, as there is insufficient evidence on these other clinical outcomes. Also, there is a wide heterogeneity across the studies, with differences in study designs, duration of aspirin use, and varying sample sizes. Wide heterogeneity across studies also prevents us from making firm conclusions regarding the beneficial effects of aspirin on all-cause mortality or mixed findings regarding the effect of aspirin or clopidogrel on dyspnea or myocardial infarction. Moreover, clinical profile and risk stratification need to be performed to identify patients with a specific clinical profile of COPD patients who can benefit from aspirin or clopidogrel. 

## Figures and Tables

**Table 1:** Characteristics of the studies included in meta-analysis (*n* = 12).

Study	Year	Country	Study Design	Sample Size	Patients	Intervention	Control	Intervention Duration	Outcome
Soyseth et al., 2007 [18]	2007	Norway	Hospital-based study	854	COPD	Aspirin	No Aspirin	1.9 years	Mortality
Short et al., 2011 [19]	2011	Scotland	Hospital-based study	5977	COPD	Aspirin	No Aspirin	4.35 years	Mortality
Ekstrom et al., 2013 [14]	2013	Sweden	Time-dependent analysis	2249	COPD	Aspirin	No Aspirin	1.1 years	Mortality
Harrison et al., 2014 [15]	2014	UK	Observational cohort study	1343	COPD	Aspirin	No Aspirin	1 year	Mortality
Andell et al., 2015 [20]	2015	Multi-country	Double-blinded RCT	1085	Diagnosed with COPD and Acute coronary syndrome	Clopidogrel	Ticagrelor	one year	Death from any cause, major bleeding, dyspnea
Campo et al., 2017 [21]	2017	Italy	RCT	46	COPD	Aspirin	No Aspirin	One month	Dyspnea
Gote et al., 2018 [22]	2018	United States	Retrospective cohort study	206,686	Acute exacerbation of COPD	Aspirin	No Aspirin	3 months	In-hospital death, length of hospital stays, invasive mechanical ventilation
Fawzy et al., 2018 [23]	2018	United States	Observational cohort study	1698	COPD	Aspirin	No Aspirin	One month	Dyspnea
Rodríguez-Mañero et al., 2019 [24]	2019	Spain	Prospective analysis	937	COPD	Aspirin and Clopidogrel	No treatment	NR	Mortality
Ellingsen et al., 2020 [25]	2020	Sweden	Observational retrospective cohort study	17,745	COPD	Aspirin	No Aspirin	3.62 years	Mortality
Yu et al., 2022 [26]	2022	China	Retrospective cohort study	42,728	COPD	Aspirin	No Aspirin	2.6 years	Death, major bleeding
Li et al., 2023 [27]	2023	China	Retrospective cohort study	56,660	COPD	Aspirin	No Aspirin	NR	MI, dyspnea, death
